# Pied de Charcot: un diagnostic à ne pas méconnaitre

**DOI:** 10.11604/pamj.2015.22.83.7652

**Published:** 2015-10-01

**Authors:** Naziha Khammassi, Youssef Kort

**Affiliations:** 1 Faculté de médecine de Tunis,Service de médecine interne, Hôpital Razi, la Manouba 2010, Tunisie

**Keywords:** pied diabétique, pied de Charcot, ostéoarthropathie diabétique, diabetic foot, Charcot foot, diabetic osteoarthropathy

## Image en medicine

L'ostéoarthropathie diabétique est une complication qui se manifeste, au stade précoce, par une inflammation localisée du pied ou de la cheville secondaire à une ostéolyse d'origine inflammatoire et neuropathique. En l'absence de décharge, elle conduit à de sévères atteintes osseuses, responsables d'une forte morbi-mortalité. Les examens complémentaires permettent d'évaluer l'étendue et la sévérité de l'atteinte. Le but du traitement est de limiter la déformation par l'immobilisation plâtrée et la décharge. La chirurgie est recommandée pour éviter la survenue d'ulcérations secondaires aux déformations. Patient âgé de 62 ans suivi pour diabète de type II évoluant depuis 17 ans, avec un équilibre glycémique sous optimal (HbA1c oscillant entre 8 et 9%), insuliné depuis 2003. Son diabète était au stade de complications dégénératives à type du pied diabétique avec amputation des deux premiers orteils gauches en 2010 et du gros orteil droit en 2014. A l'examen clinique on notait un pied droit déformé, indolore, tuméfié dans sa globalité avec une augmentation importante du son volume et un lymphœdème de la jambe. Par ailleurs il n'y avait pas de signes inflammatoires ni fluctuation. La biologie montrait une glycémie élevée (entre 3 et 4 g/l) sans syndrome inflammatoire associé. L'imagerie par résonance magnétique objectivait une importante destruction osseuse. Les causes infectieuses, tumorales et dégénératives ont été éliminées par l'interrogatoire, l'examen clinique et les examens radiologiques. Le diagnostic du pied de Charcot a été retenu et devant l'atteinte importante de l'articulation une amputation de la jambe a été indiquée après équilibration de son diabète.

**Figure 1:**
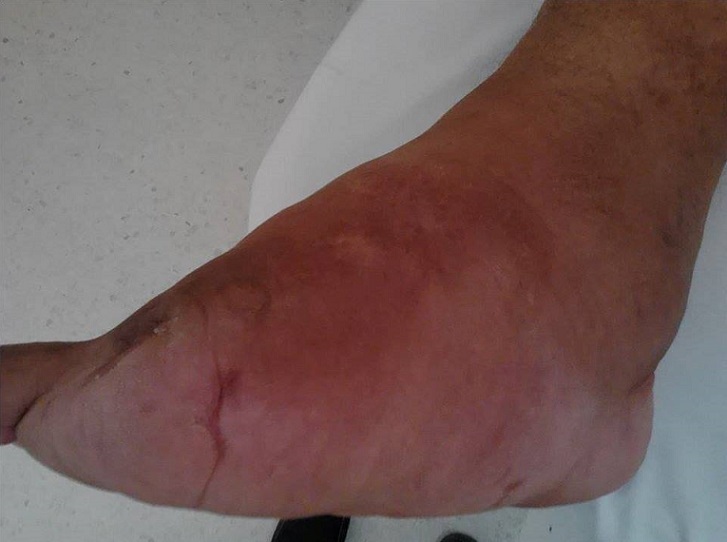
Pied déformé, tuméfié dans sa globalité avec un lymphœdème de la jambe

